# The Latest Battles Between EGFR Monoclonal Antibodies and Resistant Tumor Cells

**DOI:** 10.3389/fonc.2020.01249

**Published:** 2020-07-24

**Authors:** Wen-Qi Cai, Li-Si Zeng, Li-Feng Wang, Ying-Ying Wang, Jun-Ting Cheng, Ying Zhang, Zi-Wen Han, Yang Zhou, Shao-Li Huang, Xian-Wang Wang, Xiao-Chun Peng, Ying Xiang, Zhaowu Ma, Shu-Zhong Cui, Hong-Wu Xin

**Affiliations:** ^1^Laboratory of Oncology, Center for Molecular Medicine, School of Basic Medicine, Health Science Center, Yangtze University, Jingzhou, China; ^2^Department of Biochemistry and Molecular Biology, Health Science Center, School of Basic Medicine, Yangtze University, Jingzhou, China; ^3^State Key Laboratory of Respiratory Disease, Affiliated Cancer Hospital & Institute of Guangzhou Medical University, Guangzhou, China; ^4^Department of Gynaecology and Obstetrics, Lianjiang People's Hospital, Lianjiang, China; ^5^Department of Clinical laboratory, Lianjiang People's Hospital, Lianjiang, China; ^6^Department of Laboratory Medicine, Health Science Center, School of Basic Medicine, Yangtze University, Jingzhou, China; ^7^Department of Pathophysiology, Health Science Center, School of Basic Medicine, Yangtze University, Jingzhou, China

**Keywords:** monoclonal antibodies, non-coding RNA, tumor microenvironment, epidermal growth factor receptor, resistance, exosomes

## Abstract

Epidermal growth factor receptor (EGFR) is a tyrosine kinase receptor involved in homeostatic regulation of normal cells and carcinogenesis of epithelial malignancies. With rapid development of the precision medicine era, a series of new therapies targeting EGFR are underway. Four EGFR monoclonal antibody drugs (cetuximab, panitumumab, nimotuzumab, and necitumumab) are already on the market, and a dozen other EGFR monoclonal antibodies are in clinical trials. Here, we comprehensively review the newly identified biological properties and anti-tumor mechanisms of EGFR monoclonal antibodies. We summarize recently completed and ongoing clinical trials of the classic and new EGFR monoclonal antibodies. More importantly, according to our new standard, we re-classify the complex evolving tumor cell resistance mechanisms, including those involving exosomes, non-coding RNA and the tumor microenvironment, against EGFR monoclonal antibodies. Finally, we analyzed the limitations of EGFR monoclonal antibody therapy, and discussed the current strategies overcoming EGFR related drug resistance. This review will help us better understand the latest battles between EGFR monoclonal antibodies and resistant tumor cells, and the future directions to develop anti-tumor EGFR monoclonal antibodies with durable effects.

## Introduction

Over 30 years ago, Stanley Cohen and Rita Levi-Montalcini discovered epidermal growth factors (EGF) and nerve growth factors (NGF) and won the Nobel Prize for Physiology and Medicine (1). Epidermal growth factor receptor (EGFR), also known as Her-1 or ErbB-1, the expression product of the proto-oncogene C-erbB-1, is a 170-kDa transmembrane glycoprotein composed of a single polypeptide chain. EGFR (HER1), ErbB-2 (HER2), ErbB-3 (Her3), and ErbB-4 (Her4) constitute the ErbB receptor family.

Cancers are difficult to treat due to their complexity (2–6). Members of the HER family are overexpressed, dysregulated, or mutated in many human Tumors, including colorectal, head and neck, and small cell lung cancers. As a result, EGFR has become one of the most popular cancer treatment targets (7). To date, there are two main drug types for cancer targeted therapy based on high EGFR expression: EGFR monoclonal antibodies, including the currently approved cetuximab, panitumumab, nimotuzumab, and necitumumab, and tyrosine kinase inhibitors, including afatinib, erlotinib, gefitinib, and osimertinib, which have been approved for marketing. However, as with other cancer therapeutics, these treatments lead to drug resistance (8), and only a few patients have a lasting response to currently available treatments.

In this review, we summarize the mechanisms of action of monoclonal antibody drugs targeting EGFR as well as their clinical trials and market conditions. We additionally list the latest EGFR drug resistances and comprehensively evaluate the latest strategies to overcome EGFR resistance.

## Characteristics of EGFR

**Figure 1:**
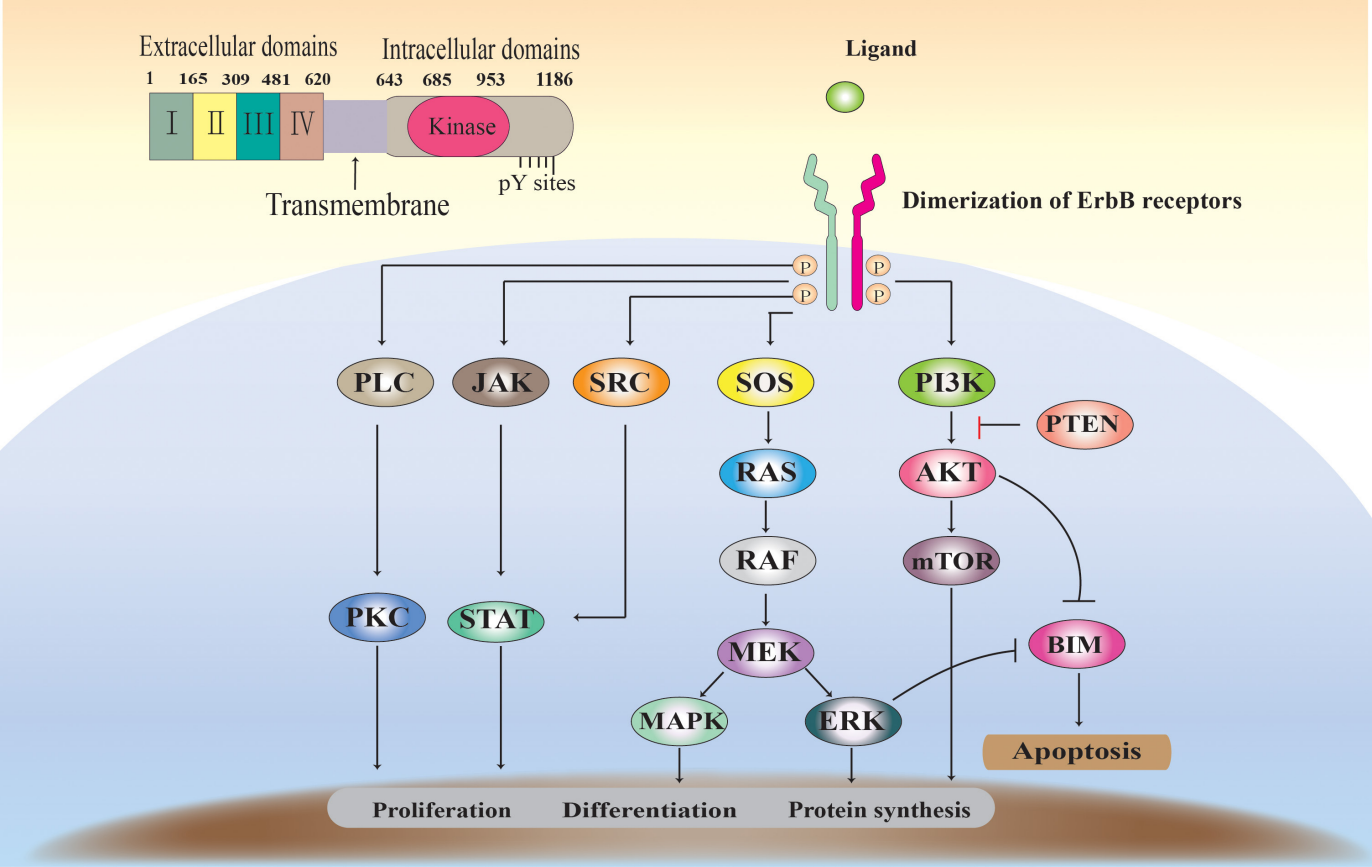
The EGFR structure, signaling pathways, and functions.

EGFR binds to its natural ligand and then form homo- or heterodimers with ErbB family members, thereby triggering activation of the downstream signaling pathway and affecting cell differentiation and proliferation. As the 60 receptor protein tyrosine kinases (RTKs) found in the human genome, EGFR primarily have extracellular ligand-binding, transmembrane and intracellular kinase regions (Figure 1) (9, 10). The extracellular domain can be divided into four sub-structures. The extracellular domain can be divided into four sub-structures. Domains I and III can to bind ligands and have a β-helical fold: Two cysteine-rich regions, domains II and IV, are responsible for the opening of the receptor dimerization interface. The transmembrane domain contains an alpha helix transmembrane peptide. The intracellular domain contains a 250-amino-acid conserved protein tyrosine kinase core and 229 C-tail residues to regulate tyrosine residues (11, 12).

ErbB receptors are widely expressed in various cell types. Under steady state conditions, receptor activity is effectively regulated by the ligand (13). Binding of ligands, such as EGF, to the EGFR extracellular domain induces EGFR dimerization, thereby activating EGFR tyrosine kinase activity and receptor trans autophosphorylation (14). EGFR ligand family can be divided into three groups. The first group includes the epidermal growth factor, epigen and amphiregulin, and transforming growth factor alpha, which are specialized to bind only EGFR. The second group includes betacellulin, epiregulin HB-EG, which bind EGFR and Her4. The third group includes neuregulin (NRG1-4), which is further subdivided based on their binding ability to Her3 and Her4 (NRG1 and nrG2) or only Her4 (nrG3 and nrG4) (15). The ErbB receptor (homologous and hetero- dimers), activated upon binding to a ligand, forms a signal transduction complex with a number of signaling proteins. Subsequently, at least five downstream signaling pathways (such as Ras/ERK, PI3K/Akt, and STAT) are activated, controlling cell proliferation, differentiation, apoptosis, and other forms of cell death. More importantly, EGFR overexpression (up-regulation or amplification) or mutation is usually associated with progression and resistance of epithelial tumors (16) (Figure 1).

## Anti-Tumor Mechanisms and Effects of EGFR mAbs

Currently, the anti-EGFR treatment includes monoclonal antibodies (mAbs), Tyrosine kinase inhibitors (TKIs), immune therapies using vaccines, and antisense therapies (17). However, monoclonal antibodies and TKIs exert effective anti-EGFR therapy in clinical trials. Currently, there are four major EGFR monoclonal antibodies approved for clinical usage, namely cetuximab, panitumumab, nimotuzumab, and necitumumab.

**Figure 2:**
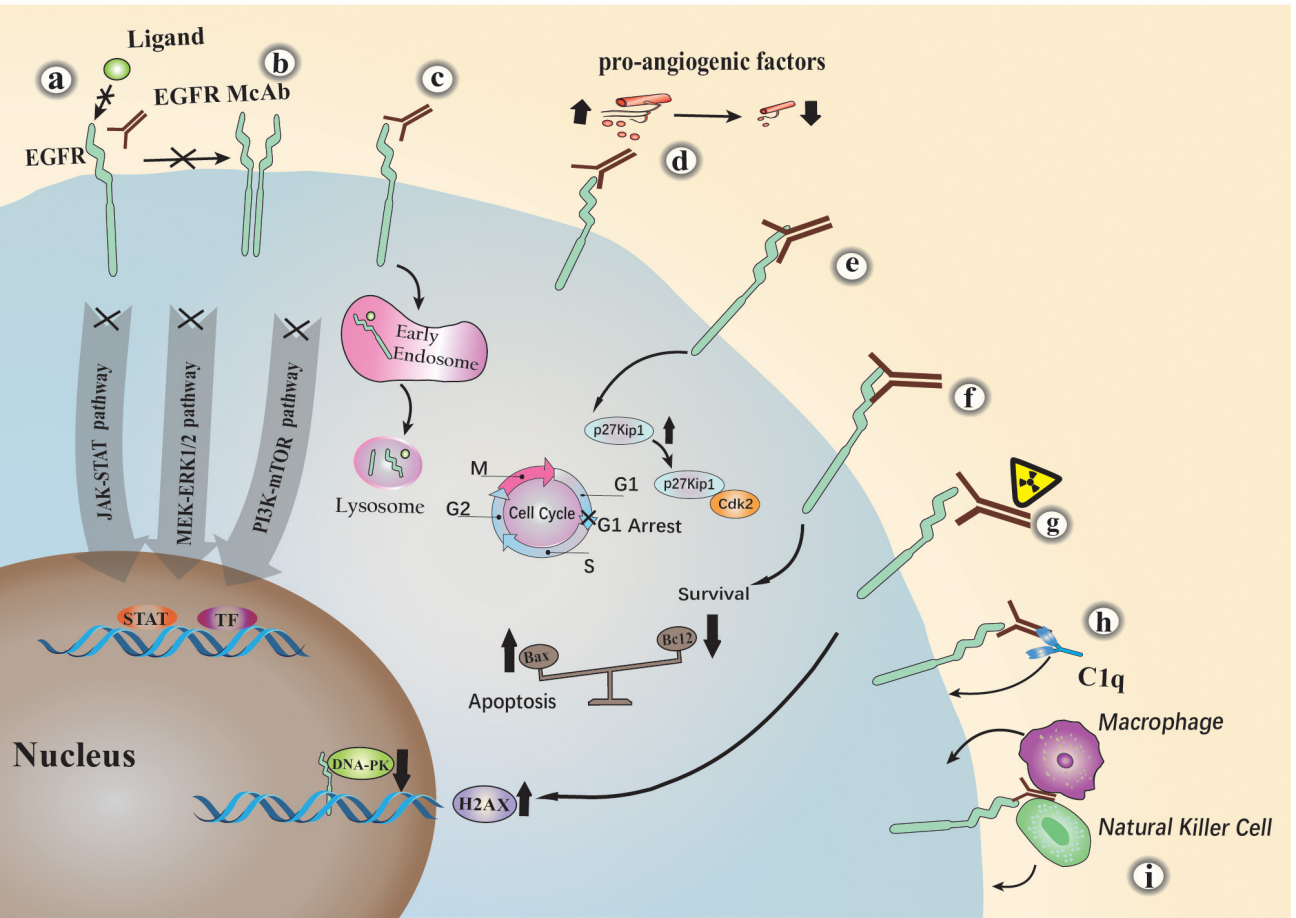
The major anti-tumor mechanisms of EGFR monoclonal antibodies. Cetuximab inhibits EGFR by the following mechanisms: (a) competitively blocking ligand binding to EGFR; (b) sterically hindering the binding of EGFR to other Her family members; (c) promoting EGFR internalization and degradation; (d) activating the complement dependent cytotoxicity (CDC) pathway; (e) causing cell cycle arrest at G1; (f) inhibiting the expression of pro-angiogenic factors; (g) promoting apoptosis; (h) natural killer (NK) cells or macrophages mediated antibody-dependent cellular cytotoxicity (ADCC); (i) increasing H2AX levels and inhibiting DNA-pk.

EGFR monoclonal antibodies exert antitumor activity by specialized structures that have different functions. The variable region fragment (FV) is an important component of a monoclonal antibody. It consists of a portion of the light and heavy chains of the antibody. This part of the structure can specifically recognize tumor receptors and cause a series of direct or indirect Tumor suppression response, taking cetuximab as an example (Figure 2). Many studies have shown that: (a). Cetuximab blocks ligand binding to EGFR by competitively binding to specific extracellular regions of EGFR, thereby inhibiting the EGFR downstream signaling (18, 19); (b). It sterically hinders the binding of EGFR to other Her family members; (c). Cetuximab inhibits EGFR downstream signaling cascade by promoting EGFR internalization and degradation (19); (d). It can also cause cell cycle arrest at G1 by increasing cell cycle inhibitor p27 kip1 and inhibiting proliferating cell nuclear antigen (PCNA) (20); (e). Tumor cells with high expression of EGFR are often accompanied by a significant increase in the level of pro-angiogenic factors, resulting in increased angiogenesis. Cetuximab can significantly inhibit the expression of pro-angiogenic factors, thereby reducing tumor angiogenesis (21–23); (f). Cetuximab or other similar anti-EGFR antibody treatments alter Bcl-2 (anti-apoptosis) and Bax (pro-apoptosis) protein balance, promoting apoptosis in tumor cells (23, 24); (g). In a recent study, cetuximab was shown to induce radiosensitization of A549 cells by increasing H2AX (H2A histone family, member X) levels and inhibiting DNA-pk (25). With the development of monoclonal antibody engineering, a variety of FV-based engineered monoclonal antibodies have been derived, such as single chain antibody fragment (scFv), Bispecific T cell Engager (BiTE) and so on. Studies have reported (26) that chimeric antigen receptor T-cell immunotherapy (CAR-T) based on scFv has significant effects in preclinical trials. In addition, oncolytic viruses armed with scFv have also shown considerable efficacy in tumor treatment (27). Fv can play a greater role in combination with other therapies due to its specific antigen recognition function, which is a key factor in targeted therapy.

The monoclonal antibody constant region FC mediates monoclonal antibody innate immunity, which mainly binds immune factors or cells in the body to exert a tumor suppressing effect. Studies have shown that (h). It inhibits tumor cell growth through the complement dependent cytotoxicity (CDC) pathway (28); (i). Cetuximab belongs to the IgG1 type and can bind to FcR on the surface of natural killer (NK) cells or macrophages by its own Fc segment, thereby producing antibody-dependent cellular cytotoxicity (ADCC) (29, 30); Pollack et al. (31) found that the direct immune effect of EGFR antibodies is related to MHC molecules. Later, some researchers compared the immune effects related to the FC region of different EGFR monoclonal antibodies. In head and neck cancer, Trivedi et al. (32) performed a side-by-side comparison of cetuximab and panitumumab. The results showed that although panitumab the degree of effective inhibition of EGFR signaling by mAb is similar to that of cetuximab, but it is less effective in mediating anti-tumor cell immune mechanisms. Similarly, cetuximab, panitumumab, and nimotuzumab The direct comparison between them was carried out by Mazora et al. (33). Although all three drugs target the EGFR receptor, they can cause different immune effects, and we can better combine drugs for these differences. Therefore, the innate immunity caused by the monoclonal antibody FC region is very important in the treatment of tumors.

Similarly, nimotuzumab and panitumumab also exert an anti-tumor effect by competitively binding to different regions of the EGFR extracellular region to inhibit downstream signaling pathways. Panitumumab is a fully human recombinant κ immunoglobulin G2 (IgG2) monoclonal antibody with a molecular weight of ~147 kD and a high-affinity specific binding to the extracellular domain of EGFR (30). Nimotuzumab (h-R3) is a humanized monoclonal antibody that inhibits the downstream cascade of EGFR signaling by specifically binding to the extracellular domain of EGFR (34). Natural killer (NK) cells are activated by nimotuzumab, after which nimotuzumab induce Dendritic cell (DC) maturation and tumor antigen (TA)-specific CD8+ T cells. HLA-I expression on tumor cells can be restored by nimotuzumab, reversing an EGFR-mediated mechanism of immune-escape of tumors (35). The extracellular region domain III of EGFR can be bound by nimotuzumab, which overlaps with the site recognized by cetuximab mAb. The difference is that nimotuzumab binds to EGFR and blocks EGF binding, but allows EGFR to adopt its active conformation, and EGFR signaling can be reduced to a ligand-independent level via nimotuzumab interference. Necitumumab (IMC-11F8) is a fully human IgG1 mAb that targets the region of EGFR extracellular domain III and blocks ligand binding to EGFR, which is currently used primarily for the treatment of non-small cell lung cancer (NSCLC) (36). Although both Necitumumab and Nimotuzumab bind to the domain III of EGFR, they bind to different sites. Nimotuzumab competitively bind to the domain III (353–358) of EGFR with ligand and Necitumumab competitively bind to the domain III (384–409) of EGFR with ligand, this structural difference may lead to differences in the efficacy and resistance of the two antibodies.

Besides the above four approved drugs, there are some other antibodies currently being tested: Mab A13, AMG595, cetuximab (Erbitux, C225), depatuxizumab (ABT 806), depatuxizumab, mafodotin, duligotuzumab (MEHD7945A, RG7597), Futuximab (Sym004), GC1118, imgatuzumab (GA201), matuzumab (EMD 72000), necitumumab (Portrazza), nimotuzumab (h-R3), anitumumab (Vectibix, ABX-EGF), zalutumumab, humMR1, and tomuzotuximab (Table 1).

**Table 1:** Complete list of EGFR monoclonal antibodies (37–40).

**Drugs**	**Mechanism of action of McAb**	**Disease**	**Antibody type**	**R&D company**	**Market status**	**Clinical trial batch number**	**Patent expiry**
Mab A13	Competitively bind to the domain III of EGFR with ligand	NR	Humanized IgG1 antibody	NR	NR	NR	NR
AMG595	Linker to the cytotoxic agent maytansinoid DM1 target EGFRvIII	Recurrent glioblastoma Multiforme	Humanized IgG1 antibody	Amgen Inc.	Clinical trials	NCT01475006	NR
Cetuximab (Erbitux, C225)	Competitively bind to the domain III (408–468) of EGFR with ligand, generate ADCC effect	HNSCC (M) Metastatic colorectal cancer (M)	Chimeric IgG1 antibody	Eli Lilly and Company, Merck KGaA, Bristol Myers Squibb Company	Marketed	NCT01012258	02/2018
Depatuxizumab (ABT 806)	Competitively bind to region of domain II with ligand and inhibits receptor dimerization	Solid tumors	Humanized IgG1 antibody	AbbVie Inc	Clinical trials	NCT01800695	NR
Depatuxizumab mafodotin	NR	NSCLC Malignant glioma Glioblastoma Multiforme	IgG1 antibody to toxin conjugate	AbbVie Inc	Clinical trials	NCT02573324 NCT02343406	NR
Duligotuzumab (MEHD7945A, RG7597)	A monoclonal antibody with dual EGFR/HER3 specificity	Colorectal cancer HNSCC NSCLC	Humanized IgG1 antibody	Hoffmann-La Genentech Inc, F. Roche Ltd.	Clinical trials	NR	09/2031
Futuximab (Sym004)	Competitively bind to the domain III of EGFR with ligand	Colorectal cancer HNSCC NSCLC	Mixture of two chimeric IgG1 antibodies	NR	NR	NR	05/2028
GC1118	Competitively bind to the domain III (350–360) of EGFR with ligand	Colorectal cancer Gastric cancer Solid tumors	Humanized IgG1 antibody	Green Cross Corporation	Clinical trials	NCT03618667	12/2029
Imgatuzumab (GA201)	The Fc region of GA201 is glycosyl-modified to contain bisected non-fucosylated carbohydrates to enhance binding to FcγRIIIA	Squamous Cell Carcinoma Head and Neck	Humanized IgG1 antibody	Roche	Clinical trials	NCT01046266	NR
Matuzumab (EMD 72000)	Competitively bind to the domain III (460–461) of EGFR with ligand	INACTIVE	Humanized IgG1 antibody	EMD Serono Inc., Merck KgaA	INACTIVE	NCT00215644	09/2013
Necitumumab (portrazza)	Competitively bind to the domain III (384–409) of EGFR with ligand	NSCLC (M) Solid tumors	Humanized IgG1 antibody	Eli Lilly and Company	Marketed	NCT01624467	03/2025
Nimotuzumab (h-R3)	Competitively bind to the domain III (353–358) of EGFR with ligand	HNSCC (M) Metastatic pancreatic Cancer Esophageal cancer Gastric cancer	Humanized IgG1 antibody	InnoMab Pte. Ltd., Biocon Ltd	Marketed	NCT00369447	11/2015
Panitumumab (Vectibix, ABX-EGF)	Competitively bind to the domain III (386–391) of EGFR with ligand	Metastatic colorectal Cancer (M) Solid tumors	Humanized IgG2 antibody	Amgen Inc., Takeda Pharmaceutical Company Ltd.	Marketed	NCT00842257	04/2020
Zalutumumab	NR	NR	Humanized IgG1 antibody	Genmab A/S	Clinical trials	NCT01054625	NR
HumMR1	The MR1 murine antibody was subjected to humanization through CDR grafting method	NR	Chimeric anti-EGFR monoclonal IgG1 antibody	NR	NR	NR	NR
Tomuzotuximab	IgG1 sugar engineered mAb	Solid tumors	Cetuximab is enhanced by IgG1 glycosylation to enhance ADCC effect	Glycotope GmbH	Clinical trials	NTC01222637	NR

*EGFR, epidermal growth factor receptor; EGF, epidermal growth factor; M, marketed; HNSCC, head and neck squamous cell carcinoma; NR, not reported; NSCLC, non-small cell lung cancer; DM1, a cytotoxic agent; ADCC, antibody-dependent cell-mediated cytotoxicity; HER3, Receptor tyrosine-protein kinase erbB-3; CDR, complementarity-determining region; mAB, monoclonal antibody*.

We summarized all the EGFR monoclonal antibody drugs described above (Table 1). These monoclonal antibodies are basically the IgG type, primarily IgG1. Unlike IgG2 type antibodies, the specific response functions of IgG1 mainly include ADCC and CDC (41). Panitumumab is a human IgG2 isotype, which is often considered to have limited immune effector functions (42). Most of these antibodies are humanized or chimeric antibodies with reduced immunogenicity and antigen affinity. These EGFR monoclonal antibodies can have different anti-cancer characteristics depending on their Fv and Fc regions. The drug patents of panitumumab, necitumumab, matuzumab, and cetuximab have expired or are about to expire.

### Pre-clinical Studies and Clinical Trials of EGFR mAbs Available on the Market

EGFR is involved in the growth and metastasis of a variety of epithelial malignancies, and EGFR inhibitors have been used as a routine treatment in clinical oncology. Here, key and new clinical trials are collated (8) (Table 2).

**Table 2:** Clinical trials of the approved EGFR antibody drugs.

**Tumor type**	**Drug X**	**Combination therapy drugs**	**Route, dose**	**Comparison (medication group and control group)**	**Efficacy (PR, OS, PFS et), survival benefit = months**	**Safety (list grade III and IV adverse events)**	**Phase I, II, III (*n*)**	**References/** **clinical trials**
Metastatic colorectal cancer	Cetuximab	Irinotecan	Initially 400 mg/m^2^ followed by weekly 250 mg/m^2^	Cetuximab combined with irinotecan (218) vs. irinotecan (111)	PR 22.9 vs. PR 10.8%	Grade III and IV adverse events 65.1 vs. 43.5%	Phase II (329)	(43)
Colorectal cancer	Cetuximab	BSC	Initially 400 mg/m^2^ to weekly infusions at 250 mg/m^2^	Cetuximab plus BSC (287) vs. BSC (285)	OS = 6.1 vs. 4.6 KRAS wild type OS = 9.5 vs. 4.8	Grade III and IV adverse events 78.5 vs. 59.1%	Randomized Phase III (572)	(44)
Head and neck squamous cell cancer	Cetuximab	XRT	400 mg/m^2^ initial dose to followed weekly doses at 250 mg/m^2^	RT (213) vs. RT plus Cetuximab (211)	OS = 29.3 vs. 49.0	Received cetuximab had a greater number of grade 3 and 4 infusion reactions (3%)	Phase III (424)	(45)
Head and neck squamous cell carcinoma	Cetuximab	Panitumab	Cetuximab 2.5, 25, 62.5 mg/m^2^ panitumumab-0.06, 0.5, and 1 mg/kg,.	Cetuximab (*n* = 12) vs. panitumab (*n* = 15)	NR	Cetuximab-IRDye800CW and panitumumab-IRDye800CW grade 1 adverse events, respectively, is 15 and 1	Phase I (27)	(46)
Advanced NSCLC	Cetuximab	Carboplatin and paclitaxel	250 mg/m^2^ weekly after loading dose	Cetuximab vs. control	Median OS = 5.4 vs. 4.8	210 [37%] in the cetuximab group vs. 158 [25%] in the control group	Phase 3 (277)	(47)
SCCHN	Cetuximab	Nivolumab	250 mg/m^2^ weekly after loading dose	Cetuximab + IC vs. Nivolumab+ Cetuximab	OS = 5.1 vs. 7.1	Favored nivolumab vs. IC	Phase 3 (361)	NCT02105636
Metastatic colorectal cancer	Panitumumab	BSC	Panitumumab 6 mg/kg every 2 weeks	Panitumumab plus BSC (231) vs. BSC (232)	PR = 8 vs. 0%	Skin Toxicity 3/414 vs. 0%	Phase III (463)	(48)
Metastatic colorectal cancer	Panitumumab	FOLFOX4	Intravenously (IV) over 1 h at 6 mg/kg every 2 weeks on day	Panitumumab-FOLFOX4 vs. FOLFOX4 (1:1)	PFS = 9.6 vs. 8.0 OS = 23.9 vs. 19.7	Grade 3/4: 96 vs. 31%	Phase III (1,183)	(49)
KRAS wild-type biliary cancer.	Panitumumab	Cisplatin and gemcitabine	9 mg/kg BW, i.v q3w	Cisplatin and gemcitabine + panitumumab (A) vs. cisplatin + gemcitabine (B)	PFS = 54 vs. 73% OS = 12.8 months (arm A) vs. 20.1 months (arm B)	Neutropenia26 (44%) vs. 13 (47%)	Phase II (90)	(50)
Confirmed metastatic colon or rectum adenocarcinoma	Panitumumab	BSC	6.0 mg /kg	Panitumumab with BSC, *n* = 142; vs. BSC, *n* = 128	PFS = 5.2 vs. 1.7 OS = 10.2 vs. 7.4	PAN WITH BSC 46.4% vs. BSC18.7	Phase III (270)	(51)
HNSCC	Nimotuzumab	Cisplatin	Nimotuzumab (200 mg/week)	CRT + nimotuzumab (20) vs. CRT (20) vs. RT + nimotuzumab (17) vs. RT groups (19)	CR = 90 vs. 70 vs. 70.59 vs. 31.01% PR = 10 vs. 0 vs. 5.88 vs. 5.26%	Grade 3/4: 55 vs. 25 vs. 59 vs. 84%	Phase IIb (92)	(52)
Nasopharyngeal carcinoma.	Nimotuzumab	Chemoradiotherapy	200 mg, iv weekly for 7 courses	Single-arm treated with induction chemotherapy, sequential Nimotuzumab plus concurrent chemoradiotherapy	OS = 85.6% LRC = 97.8% PFS = 79.5%	Neutropenia (35.5) Thrombocytopenia (17.7)	Phase II (45)	(53)
Advanced esophageal squamous cell cancer	Nimotuzumab	Paclitaxel and cisplatin	Nimotuzumab 200 mg weekly	Single arm	RR = 51.8% DCR = 92.9% OS = 14.0 months	Alopecia (78.6%) Neutropenia (46.4%), nausea (48.3%)	Phase II (56)	(54)
Non-small-cell lung cancer	Necitumumab	Paclitaxel-carboplatin chemotherapy	Necitumumab 800 mg, on days 1 and 8	Neci with Pac-Carb (*n* = 110) vs. Pac-Carb (*n* = 57)	PFS = 5.4 vs. 5.6 OS = 13.2 vs. 11.2	Grade 3/4: 65.1% vs. 69.1%	Phase II (167)	(55)
Non-small-cell lung cancer	Necitumumab.	Gemcitabine and cisplatin	Administered IV on days 1 and 8 (necitumumab continuation arm)	Necitumumab Continuation (*n* = 261) vs. Gemcitabine-Cisplatin non-progressors (*n* = 215)	Median OS = 15.9 vs. 15.0 months; median PFS = 7.4 vs. 6.9 months;	185 (70.9) 114 (53)	Phase III (476)	(56)

*BSC, best supportive care; EGFR, epidermal growth factor receptor; FOLFOX, folinic acid (leucovorin), fluorouracil, oxaliplatin; FU, fluorouracil; HNSCC, head and neck squamous-cell carcinoma; NR, not reported; NSCLC, non-small-cell lung cancer; PR, Response rate; OS: Median overall survival; DCR, disease control rate; LCR, locoregional control; PFS, Median progression free survival; XRT, X-ray Radiation Therapy; RT, Radiation Therapy; OR, Overall Response; IC, Investigator's choice*.

#### Cetuximab

Cetuximab was approved by the FDA for metastatic colorectal cancer (CRC) and KRAS wild-type CRC in 2004 and 2009, respectively. A series of related clinical trials confirmed the efficacy of cetuximab. In a randomized phase II clinical trial of cetuximab and irinotecan in the treatment of irinotecan refractory metastatic CRC, EGFR-positive patients were randomized into two groups according to a 2:1 approach. One group received cetuximab alone (111 patients) and the other group received irinotecan + cetuximab (218 patients). This study confirmed that cetuximab has certain effect as the partial radiological response rate of the combination therapy and monotherapy groups were 22.9 and 10.8%, respectively. In addition, there was a significantly longer time to tumor progression in favor of the combination arm (4.1 compared to 1.5 months). The toxicity of the combination treatment group was more frequent, but its severity were similar to those expected for irinotecan alone (43). This level of radiological response has accelerated FDA approval of cetuximab in the United States. Full approval followed the demonstration that cetuximab with best supportive care was associated with an overall survival (OS) advantage when compared to best supportive care alone (57). However, the use of cetuximab's small-scale efficacy and the determination of KRAS status made its clinical significance questionable. In a retrospective analysis of tumor patients, OS after cetuximab treatment was higher in patients with KRAS wild-type tumors (9.5 vs. 4.8 months). The incidence of adverse events at grade 3 (or higher) in the cetuximab group was 78.55% compared to 59.1% in the support group alone (*P* < 0.001) (44).

Phase III or IV clinical trial was carried out comparing the efficacy of X-Ray Therapy (XRT) alone or XRT cetuximab in patients with head and neck squamous cell carcinoma. The trial (45) recruited 424 patients at multiple countries and centers in <3 years. Patients were randomly assigned to receive radiotherapy with (*n* = 211) or without (*n* = 213) cetuximab. Weekly doses of cetuximab: 400 mg/m^2^ initial dose, followed by seven weekly doses at 250 mg/m^2^. Cetuximab has significantly improved the OS. For patients treated with RT plus cetuximab, the median duration of OS was 49.0 months, but was 29.3 months for those treated with RT alone. Furthermore, the authors did not report any increase in the toxicity usually associated with radiotherapy for HNSCC. FDA approved cetuximab in combination with local or regional advanced XRT refractory HNSCC or as a platinum refractory monotherapy for recurrent or metastatic HNSCC.

Herbst et al. (47) studied the activity of cetuximab with carboplatin and paclitaxel in EGFR-FISH positive (EGFR-FISH+) patients. Progression-free survival (PFS) was not significantly different between the arms among the EGFR-FISH+ subpopulation (HR = 0.92 [0.75–1.12], *P* = 0.40). OS was not significantly different among treatment arms in the overall study population (HR = 0.93 [0.83–1.04], *P* = 0.22). In a prespecified analysis among patients with EGFR-FISH+ cancers, OS was significantly improved in the cetuximab group (HR = 0.58 [0.39–0.86], *P* = 0.01): median OS of 11.8 months (95% CI: 8.6–13.5) and 6.1 months (95% CI: 4.2–8.7) in cetuximab and chemotherapy arms, respectively. The most common grade 3–4 Adverse Events (AEs) were neutropenia (*n* = 210 cetuximab; *n* = 158 control). Grade 5 AEs occurred in 5% (*n* = 32) of cetuximab and 2% (*n* = 13) of controls.

Recently, researchers have begun to use therapeutic antibodies for surgical imaging and other related research. Gao et al. (46) studied the safety and pharmacodynamic properties of cetuximab-IRDye800CW and panitumumab-IRDye800CW. In two phase I trials, their results showed that the two drugs had similar toxicity and efficacy to the parent compound, and the experimental results indicated the therapeutic antibody can be used as a clinically relevant imaging agent. These studies showed the safety and efficacy of cetuximab modification, and indicate that such antibodies can be used as imaging agents.

In a phase III clinical trial, Ferris et al. (58) conducted a combination of EGFR monoclonal antibody and PD-1 monoclonal antibody. Stratification was based on previous cetuximab exposure. For patients previously receiving cetuximab, the median OS of nivolumab was 7.1 months and the median OS of IC was 5.1 months (HR, 0.84; 95% CI, 0.62–1.15). Nivolumab appeared to improve efficacy vs. IC regardless of prior cetuximab use, supporting its use in patients with R/M SCCHN with or without prior cetuximab exposure. Cetuximab regulates the immune response and may affect the efficacy of subsequent immunotherapy. The combination of cetuximab with other new drugs is worth studying to get better results.

#### Panitumumab

In a clinical study (49), 463 patients with colorectal cancer were randomly assigned to 2 treatment groups (1:1). One group was treated using the best supportive treatment (232), whereas the other group was treated with panitumumab plus best supportive care (231). The favorable effect of panitumumab plus best standard care (BSC) on PFS was evident among all patients, irrespective of age, and number of previous treatments. The FDA updated the label of panitumumab for the treatment of metastatic colorectal cancer, including information about KRAS mutations: When the KRAS mutation in codon 12 or 13 was found, panitumumab showed no therapeutic effect.

Other studies have shown that panitumumab was ineffective in patients with NRAS mutations (59). It is also approved as a first-line agent in combination with FOLFOX (49). Although EGFR is targeted like cetuximab, the mechanisms of action of the different types of EGFR antibodies, such as panitumumab (IgG2) and cetuximab (IgG1), may differ. In addition to activating the complement-dependent cytotoxicity (CDC), IgG1 EGFR monoclonal antibodies can also mediate ADCC to produce tumor killing effect.

It is not clear which drug is better, and one study showed that the activity of both drugs was similar (60). Recently, Vogel et al. (50) reported chemotherapy and panitumumab to be used in KRAS wild type biliary tumor treatment plan. They divided the patients into group A (cisplatin/gemcitabine and panitumumab) and group B (cisplatin/gemcitabine treatment without antibody). PFS rate at 6 months was 54% (group A) vs. 73% (group B). Their results show that panitumumab combined with chemotherapy does not improve OS and objective response rate of patients with KRAS wild-type advanced cholangiocarcinoma. Panitumumab may have certain effect in other types of solid tumors. In another study, Kim et al. (51) analyzed the effect of panitumumab treatment based on RAS and v-Raf murine sarcoma virus oncogene homology B (BRAF) mutation status. The results show that panitumumab improves the survival of wild-type RAS mCRC.

Although panitumumab effectively inhibits EGFR signaling to a degree similar to cetuximab, it is less effective in mediating antitumor cell immune mechanisms. This may explain the difference in their clinical efficacy (32).

#### Nimotuzumab

Nimotuzumab (h-R3), a humanized monoclonal antibody, has been used in the treatment of HNSCC in some countries (61), and is a glioma orphan drug in the United States and the European Union. In a study (52) in which nimotuzumab was used in patients with advanced HNSCC, the nimotuzumab plus CRT group showed a good 5-year survival rate. In another phase II clinical study (53), results showed that induction chemotherapy and sequential nimotuzumab plus concurrent chemoradiotherapy yielded excellent survival benefits and tolerable toxicity.

In addition, some new combination treatments seem to have great prospects. Lu et al. (54) assessed the efficacy and safety of nimotuzumab as a first-line treatment for advanced esophageal squamous cell carcinoma (ESCC). A total of 56 patients were enrolled in the trial, and the results suggest that the new combination of nimotuzumab plus chemotherapy is a more effective first-line treatment than the standard chemotherapy regimen. Other studies have shown (33) that nimotuzumab combined with cisplatin-based chemotherapy and radiotherapy will increase the frequency of peripheral CD4 + CD39 + FOXP3 + Treg, otherwise the frequency of nimotuzumab will decrease when used as monotherapy To baseline value. Therefore, studying the relationship between immune cells and nimotuzumab is beneficial to the clinical benefit of patients.

#### Necitumumab

In 2015, necitumumab was approved by the US FDA for the treatment of refractory metastatic squamous NSC lung cancer (NSCLC). In the study by Spigel et al. (55), a total of 176 patients were enrolled and randomized to either the necitumumab group or the chemotherapy alone group. Their results show that necitumumab and chemotherapy improve survival in patients with advanced squamous NSCLC. In another study, Ciuleanu et al. (56) studied the efficacy and safety of necitumumab in patients with stage IV squamous non-small cell lung cancer. The results of the study showed that necitumumab had the same efficacy and acceptable safety compared to those of other treatments (chemotherapy). Necitumumab is now a new first-line treatment option for squamous NSCLC (62). EGFR-directed monoclonal antibody therapy remains limited. At present, research related to nizumab and other immunotherapy and targeted drugs may lead to better results for patients with NSCLC.

Hence, the currently listed drugs, mono- or combination therapies, can improve survival rate and prolong remission period. Some tumors achieved complete remission. The side effects of the drugs were also within acceptable limits. However, further clinical trials are still required to determine the therapeutic effects and side effects of these drugs. More patients may benefit from these drugs in the future.

### Clinical Trials of EGFR mAbs That Are Not in the Market

Here, we listed the clinical trials in which the pre-market EGFR antibodies are being tested for their efficacy and side effects (Table 3). Duligotuzumab (MEHD7945A) is a dual EGFR/Her3 inhibitory antibody while Sym004 is a 1:1 mixture of two recombinant mAbs (futuximab and modotuximab), which bind specifically to non-overlapping epitopes located in the extracellular domain (ECD) of the EGFR. Depatuxizumab competitively binds to region of domain II with ligand and inhibits receptor dimerization. Depatuxizumab mafodotin is an Anti-EGFR Antibody Drug Conjugate. Several other antibodies (matuzumab; nimotuzumab; zalutumumab) can be competitively bind to the domain III of EGFR with ligand, but their binding sites are different.

**Table 3:** Clinical trials of the un-approved EGFR antibodies.

**Tumor type**	**Drug X**	**Combination therapy drugs**	**Route, dose**	**Comparison (medication group and control group)**	**Efficacy (n/N, % of CR, PR, SD, NR), survival benefit = months**	**Safety (list grade III and IV adverse events)**	**Phase I, II, III (*n*)**	**References/** **clinical trials**
Recurrent glioblastoma	Depatuxizumab mafodotin	Temozolomide	Depatux-m (0.5–1.5 mg/kg)	Single arm increments	OR was 14.3%, PFS was 25.2%, and OS was 69.1%.	Grades 3/4 AEs occurring in 22% of patients	Phase I multicenter trial (60)	(63)
Advanced solid tumors	Depatuxizumab (ABT-806)	NR	2 mg/kg every other week (eow) to escalate to 6, 12, 18, and 24 mg/kg eow.	Single arm increments	Median time to progression was 55 vs. 43 days. No OR occurred	Grade 3/4 AEs *n* = 11 (22%)	Phase I (49)	(64)
Squamous cell carcinoma of the head and neck	Duligotuzumab	Cetuximab	Duligotuzumab (1,100 mg IV, q2w)	Both arms (duligotuzumab vs. cetuximab 59:62)	PFS was 4.2 vs. 4.0 months; HR: 1.23, OS was 7.2 vs. 8.7 months; HR 1.15 and ORR (12 vs. 14.5%)	Duligotuzumab vs. cetuximab, and GI disorders (17 vs. 7%), infections (22 vs. 11.5%)	Phase II (121)	(65)
Metastatic colorectal cancer	Sym004 (futuximab + modotuximab)	BSC or 5-FU or Capecitabine	A: Sym004 (12 mg/kg) B: Sym004 (9/6 mg/kg)	A: Sym004 (12 mg/kg) B:Sym004 (9/6 mg/kg) C: Standard of Care	OS = 7.9 vs. 10.3 vs. 9.6 CR = 0 (0) vs. 0 (0) vs. 1 (1.2) PR = 11 (13.3) vs. 8 (9.3) vs. 1 (1.2)	A: 58 (69.9%) vs. B: 41 (48.8%) vs. C: 9 (11.5%)	Phase II (254)	NCT02083653
Head and neck squamous cell carcinoma	Imgatuzumab (GA201)	Cetuximab	Imgatuzumab (700 mg) or (1,400 mg)	Imgatuzumab (700 mg) vs. Imgatuzumab (1,400 mg) vs. Cetuximab	Downregulation of EGFR−35% [700 mg]; −42% [1,400 mg]; −21% [cetuximab]	10 (48%) vs. 14 (70%) vs. 10 (56%)	*n* = 59	(66)
Advanced gastric and esophagogastric adenocarcinomas	Matuzumab (EMD 72000)	5-fluorouracil, leucovorin and cisplatin (PLF)	400 mg matuzumab in combination with PLF or 800 mg matuzumab	400 mg dose *n* = 7; 800 mg dose *n* = 8	The best confirmed overall response rate was 26.7%	0 (0) vs. 2 (13.3%)	Phase I (15)	(67)
Advanced non-small cell lung cancer	Nimotuzumab (h-R3)	Gefitinib	200 mg, i.v. weekly	Nimotuzumab plus gefitinib (78) vs. gefitinib alone (77)	PR = 13 (16.7%) vs. 17 (22.1%) SD = 29 (37.2%) vs. 33 (42.9%) OS = 14.0 (9.7–18.2) vs. 13.5 (11.3–15.7)	11 (14.3%) vs. 12 (15.6%)	Phase II (155)	(68)
Squamous-cell carcinoma of the head and neck	Zalutumumab	Best supportive care	Initially 8 mg/kg and followed doses of 4 mg/kg	Zalutumumab plus BSC (191) vs. BSC alone (95)	OS = 6.7 (5.8–7.0) vs. 5.2 (4.1–6.4)	(39 [21%] vs. 0)	Phase III (286)	(69)
Solid tumors	Tomuzotuximab	NR	Weekly (12–1,370 mg) or 2-weekly (990 mg)	A three-plus-three dose escalation design.	12 SD, 1 PR, 1 CR, and 2 prolonged control of their non-measurable disease.	Infusion-related reactions 3 grade, 12%	Phase I (41)	(70)

*BSC, best supportive care; FOLFOX, folinic acid (leucovorin), fluorouracil, oxaliplatin; FU, fluorouracil; HNSCC, head and neck squamous-cell carcinoma; NR, not reported; NSCLC, non-small-cell lung cancer; PR, Response rate; OS, Median overall survival; PFS, Median progression free survival; DCR, disease control rate; CR, complete response; XRT, X-ray Radiation Therapy; RT, Radiation Therapy*.

Depatuxizumab mafodotin (depatux-m) as an antibody drug conjugate in an open-label research treatment of 60 patients with recurrent glioblastoma showed preferable objective response rate, PFS, and OS of 14.3, 25.2, and 69.1%, respectively (63). Depatuxizumab is mainly used for solid tumors in which EGFR is highly expressed. In a phase I clinical study, the median time to progression of 49 glioblastoma patients was 55 days and glioblastoma patients in control patients was 43 days. Depatuxizumab was administered at a dose of 2 mg/kg every other week (eow) and was extended to 6, 12, 18, 24 mg/kg (eow), with about grade 3/4 AEs in 11 patients. A total of 14 patients died during the study due to disease progression. No Overall Response (OR) occurred (64).

The efficacy of duligotuzumab (MEHD7945A) and cetuximab was evaluated in head and neck squamous cell carcinoma in a randomized phase II study. Cetuximab and duligotuzumab were comparable in PFS (4.0 vs. 4.2 months), OS (8.7 vs. 7.2 months), and objective response rates (14.5 vs. 12%), contributing to different rates of serious AEs (29.5 vs. 41%). These results indicate that HER3 inhibition has a comparable effects with EGFR inhibition (65).

Sym004 is a mixture of antibodies directed against EGFR, which consists of Futuximab and Modotuximab. A phase II clinical study of this cancer, included 254 patients divided into 3 groups: group A sym004 (12 mg/kg), group B sym004 (9/6 mg/kg), and group C as the standard treatment group. In the three groups, the OS was 7.9 vs. 10.3 vs. 9.6 months, respectively, and the PR was 13.3 vs. 9.3 vs. 1.2% (NCT02083653). Temam et al. (66) showed that, in patients with advanced head and neck cancer, one patient achieved “pathological” complete remission, and more than half of the other patients showed some efficacy when Sym004 was used.

A study found that the overall response rate of matuzumab with other chemotherapy drugs in first-line treatment in EGFR-positive patients with advanced gastric cancer and adenocarcinoma of the esophagogastric was 26.7%. The results show that the matuzumab, combined with 5-fluorouracil, leucovorin, cisplatin (PLF) is an acceptable treatment plan (67).

In a phase II study, nimotuzumab plus gefitinib and gefitinib alone after platinum-based chemotherapy Were tested in 155 advanced NSC lung cancer patients. Their OS and PR were 14.0 vs. 13.5 months and 16.7 vs. 22.1%, respectively, and the toxicity of the two groups did not significantly differ (68).

After the failure of platinum-based chemotherapy in squamous cell carcinoma patients, they were further divided into two groups: zalutumumab plus optimal supportive care and optimal supportive care, and their OS rates were 6.7 and 5.2 months, respectively (69).

## Mechanisms of EGFR Antibody Resistance

**Figure 3:**
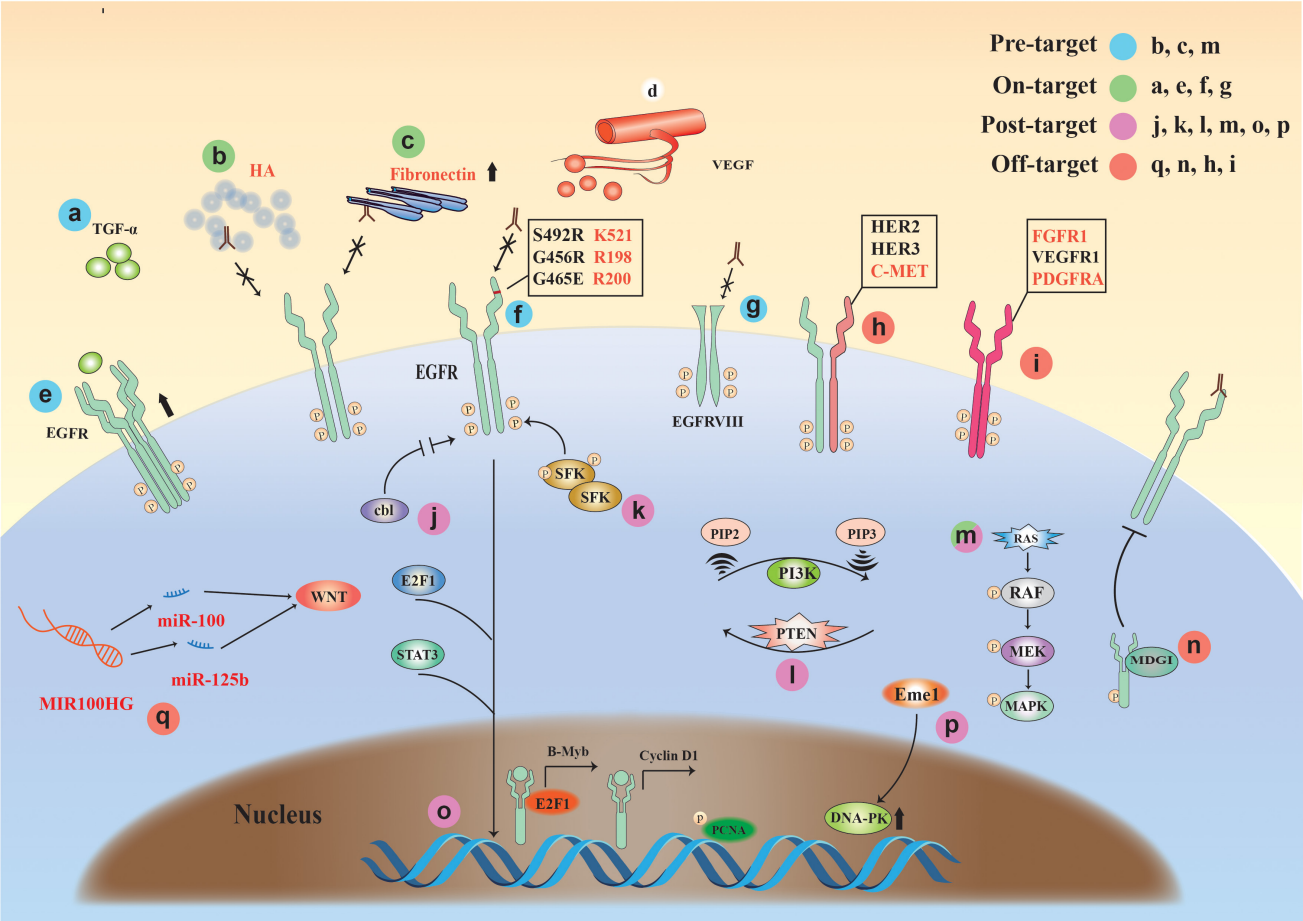
The drug resistance mechanisms of EGFR monoclonal antibodies. Four classifications: (1) Pre-target: b, c, and m (KRAS mutation, the tumor microenvironment); (2) On-target: a and e (high expression of EGFR or ligand), f and g (mutation and deletion of extracellular domain binding site); (3) Post-target: j, k, l, m, o, and p (EGFR downstream signaling pathways); (4) Off-target: q, n, h, and I (Drug resistance factors associated with non-EGFR signaling molecules or receptors).

Here, we comprehensively summarize the drug resistance mechanisms of EGFR monoclonal antibodies and classified the complex mechanisms, for the first time, into the following four classes (Figure 3): (1) Pre-target, drug resistance factors before the binding of monoclonal antibody to EGFR receptor (KRAS gene mutation, the influence of tumor microenvironment); (2) On-target, the binding of monoclonal antibody to EGFR drug resistance factors (high expression of EGFR or ligand, mutation and deletion of extracellular domain binding site); (3) Post-target, resistance mechanisms downstream of EGFR monoclonal antibody binding to EGFR; (4) Off-target, the resistance mechanisms via other signaling molecules or receptors after monoclonal antibody-EGFR binding.

### Pre-target

The tumor microenvironment (TME) is closely related to tumor drug resistance (71). The resistance against monoclonal antibody therapy also has a strong relationship with the TME. Studies have shown that the accumulation of hyaluronic acid (HA), the main component of TME, occurs in many solid tumor types and is associated with poor prognosis and therapeutic resistance in a variety of malignancies. The results suggest that high matrix HA levels in the TME may form a barrier that inhibits the entry of EGFR MAb and NK cells, promoting EGFR resistance (72).

KRAS mutations occur in colorectal cancer and are often predictive of poor sensitivity to cetuximab or panitumumab (73, 74). Currently, KRAS mutation occurs in 30–50% of colorectal cancers (75).

### On-Target

Overexpression of EGFR has also been thought to be associated with the development of acquired resistance, but one study showed that cetuximab combined with chemotherapy improved relapse/metastatic HNSCC and KRAS wild-type mCRC regardless of tumor EGFR expression levels (76).

The results of Rajput et al. (77) showed that constitutive changes in ligand TGFα can lead to constitutive activation of EGFR, leading to tumor growth and metastasis. It was found that cetuximab resistance can be induced by activating the PI3K/Akt pathway and (78) alterations of EGFR itself [e.g., EGFR polymorphisms (79) and EGFR variant III (vIII) expression (80–82)]. In addition, Cetuximab competitively bind to the domain III (408–468) of EGFR with ligand. Therefore, the exon 12 EGFR mutation (S492R) encoding the extracellular region of EGFR will affect the binding of cetuximab to the domain III of EGFR. Panitumumab competitively binds to the domain III (386–391) of EGFR with ligand, the difference in their binding sites causes differences in drug resistance (83).

Overexpression of HER family ligands is also associated with cetuximab resistance, and the nucleus entry pathway of EGFR. The nuclear expression of EGFR can be used as a resistant prediction of cetuximab reaction (84).

### Post-target

When monoclonal antibody drugs are used, most patients will develop secondary resistance, which is associated with mutations in downstream signaling of EGFR. Studies have shown that detecting the status of the gene of phosphate and tension homology deleted on chromsome 10 (PTEN), BRAF, and EGFR can determine the sensitivity of cetuximab treatment, showing that BRAF status, EGFR amplification, and PTEN cytoplasmic expression are correlated to cetuximab resistance in wild-type KRAS metastatic colon cancer (85). Some rare mutations such as BRAF-V600E mutation might result in a less beneficial anti-EGFR monoclonal antibody treatment (86).

Wheeler et al. (87) found that Src family kinases (SFKs) were highly activated in cetuximab-resistant cells, while PI3K/AKT signaling pathways and Her3 expression levels increased. Further, SFKs play a key role in cetuximab monoclonal antibody resistance. Yang et al. (88) showed that inhibition of EGFR ubiquitination in tumor cells can alter the expression level of EGFR and regulate cell growth and survival by bypassing EGFR via other pathways (e.g., Src kinase-mediated cell signaling) and cause resistance to cetuximab.

Weinandy et al. (89) suggested that Eme1 protein is significantly increased under cetuximab resistance, which often means that treatment may fail. Mechanistically, elevated levels of Mus81/Eme1 endonuclease during cetuximab treatment promoted DNA repair and ultimately led to tumor cell growth.

### Off-Target

Long non-coding RNA (lncRNA) has been reported to have a close relationship with tumors. As a class of gene regulators, lncRNA also has an important relationship with EGFR monoclonal antibody resistance. Lu et al. (90) found that the long-chain non-coding RNA MIR100HG produced miR-100 and miR-125b, which synergistically inhibited five Wnt/β-catenin-negative modulators, resulting in increased Wnt signaling, leading to cetuximab resistance. Wnt inhibition in mAb-resistant cells restored cetuximab reactivity. Peng et al. (91) found that lncRNA POU5F1P4 down-regulation promoted cetuximab resistance in mCRC. In another study, inhibition of lncRNA LINC00973 attenuated cetuximab resistance and was associated with glucose metabolism (92). The results of a study by Li et al. (93) suggest that the expression of lncRNA H19 and MALAT1 may play a role in the resistance of cetuximab in mCRC. In addition to lncRNA, other types of RNAs are also associated with EGFR monoclonal antibody resistance (94).

Studies have shown (8, 87) that when EGFR monoclonal antibody binds to EGFR, it will increase the heterodimerization of EGFR and other receptors to activate downstream signaling pathways, including Her2, Her3, and C-met. Other studies have shown (95, 96) that even if the EGFR signal is inhibited by monoclonal antibody drugs, other receptors on the tumor cell membrane (FGFR1, PDGFRA, or VEGFR1) can be overexpressed. Both of these conditions will produce resistance to EGFR monoclonal antibody drugs. In addition, milk-derived growth inhibitor (MDGI) is a small cytoplasmic protein that plays an important role in the differentiation of epithelial cells. Studies have shown that MDGI expression can affect the transmission of EGFR signaling and cause cetuximab resistance (96). A recent article reported that exosomes facilitate cetuximab resistance through the PTEN/Akt pathway in cetuximab-sensitive cancer cells (97).

With the continuous deepening of research in oncology, more and more factors related to drug resistance have been discovered. At present, tumor microenvironment, non-coding RNA, exosomes are the focus of attention. Sidaway (98) studies show that: Cetuximab resistance occurs not only through Darwinian acquisition of RAS–RAF pathway mutations, but also via microenvironmental plasticity. The strong effect of cetuximab on the immune landscape shows that systemic therapies can change immune infiltrates quite dramatically. Lu et al. (90) studies show that non-coding RNA is related to resistance to EGFR treatment. *De novo* and acquired resistance, which are largely attributed to genetic alterations, are barriers to effective anti-epidermal-growth-factor-receptor (EGFR) therapy. These findings identify a clinically actionable, epigenetic cause of cetuximab resistance.

The composition of exosomes is more complex, which contains a variety of biological macromolecules, such as: nucleic acids (double-stranded DNA and various RNA subtypes), proteins and lipids. These molecules are carried into the blood circulation by exosomes and are then taken up by target cells, thereby regulating target cell gene expression and cell function. In addition, exosomes-related miRNAs, as short single-stranded and non-coding RNA molecules, regulate the expression of oncogenes or tumor suppressor genes and participate in cell differentiation, apoptosis, and cell signal transduction (99). Studies have shown that exosomes can affect the formation of tumor microenvironment, enhance the ability of tumor cells to invade and metastasize, mediate tumor immunosuppression, and participate in tumor chemoradiotherapy resistance to promote the development of tumors (100).

In conclusion, non-coding RNA, tumor microenvironment and exosomes are closely related to EGFR monoclonal antibody resistance. Based on recent research, it is expected that effective measures will be developed that can be applied to clinical diagnosis and treatment.

## Conclusions and Perspectives

We summarize recently completed and ongoing clinical trials of the classic and new EGFR monoclonal antibodies. More importantly, according to our new standard, we re-classify the complex evolving tumor cell resistance mechanisms, including those involving non-coding RNA, tumor microenvironment and exosomes against EGFR monoclonal antibodies. Studies have shown that in KRAS wild-type CRC patients, the final response to EGFR monoclonal antibody treatment is only about 15% (96, 101). However, EGFR mAbs remain one of the main approaches for anti-cancer treatments. In recent years, research has been conducted to overcome these resistance mechanisms in the context of alterations of EGFR [e.g., EGFR polymorphisms (79), EGFR nuclear internalization, EGFR variant III [vIII] expression] (80, 81). In a recent study, the anti-EGFR antibody cocktail Sym004 was used for successful treatment in the context of CRC associated with EGFR extracellular domain mutation-mediated cetuximab resistance (102). Monoclonal antibody MM-151 binds to multiple regions of the EGFR extracellular domain, thereby inhibiting the transmission of mutant EGFR signaling (103). In another study, cetuximab was glycosylated to have a higher affinity for FcγRIIIA on human immune effector cells, further enhancing ADCC activity. Clinical studies have shown that glycosylation-modified antibodies restore sensitivity to EGFR targeting in HNSCCs expressing EGFRK521 variants (79). In addition, the deletion of exons 2–7 of EGFRvIII produced a unique acid site, which makes the development of EGFRvIII specific MAbs a possibility. These antibodies have been used to detect EGFRvIII and to overcome EGFR resistance (104, 105). Some tumor-related predictors can foresee patterns of drug sensitivity. KRAS status is a predictive gene for cetuximab or panitumumab in metastatic colorectal cancer and is a routine test before administration (64, 106). A recent study showed that Insulin receptor substrate 2 (IRS2) mutations can be used as a predictor of anti-EGFR sensitivity when treating cancers with high EGFR expression (96). Similarly, the amplification of the EGFR gene (EGFRAMP) has also been reported to increase the sensitivity of treatment (107). However, with the advent of the era of precision medicine, targeted drugs have not achieved long-term response, and new drug resistance mechanisms have emerged. Doctors are looking for different strategies to cope with new drug resistance mechanisms and achieve long-term disease control (108). The treatment of single-gene monotherapy has become a thing of the past, and the era of multidimensional treatment has arrived (109).

It seems that there is still a long way to go before EGFR monoclonal antibody drugs are fully efficient. In recent years, with the emergence of CAR-T, oncolytic viruses (6, 110), and other therapies (108, 111–113), scFv has been used to target EGFR. scFv is smaller but retains the variable region function of the mAb (114). CAR consists of three regions: the extracellular, transmembrane, and intracellular domains (111). There seems to be a link between the extracellular antigen recognition and intracellular signaling domains for activating T cells. In turn, a series of tumor-killing effects are produced. Part of the extracellular domain of CAR that binds to the antigen can be the scFv to EGFR (115). In a recent study, a peptide against panErbB-CAR is now in clinical trial in HNSCC (116). In another study, the EGFR-retargeted oncolytic virus containing an anti-EGFR scFv proved to be effective in an orthotopic mouse model of primary human glioma (117). Thus, the scFv produced by the variable region of EGFR monoclonal antibodies could potentially be a significant component in the future treatment of cancers. Getting the optimal effects with minimal adverse effects will be the future development direction in the continued battles between EGFR monoclonal antibodies and resistant tumor cells.

## Consent to Publication

We declare that all authors agreed to publish the manuscript at this journal based on the signed Copyright Transfer Agreement, and followed publication ethics.

## Author Contributions

W-QC, ZM, and H-WX: contributed to this paper with the design. W-QC, L-SZ, and J-TC: literature search. W-QC, Y-YW, Z-WH, and YZ: drafting. W-QC, YZ, S-LH, L-FW, S-ZC, H-WX, X-WW, X-CP, and YX: revision. L-FW, S-ZC, and H-WX: editing. H-WX: final approval. All authors contributed to the article and approved the submitted version.

## Conflict of Interest

The authors declare that the research was conducted in the absence of any commercial or financial relationships that could be construed as a potential conflict of interest.
